# Stiff morphing composite beams inspired from fish fins

**DOI:** 10.1098/rsfs.2023.0072

**Published:** 2024-06-07

**Authors:** Saurabh Das, Prashant Kunjam, Baptiste Moling, Tian Gao, Francois Barthelat

**Affiliations:** ^1^ Department of Mechanical Engineering, University of Colorado, 427 UCB, 1111 Engineering Dr, Boulder, CO 80309, USA; ^2^ Ecole Polytechnique, Route de Saclay, Palaiseau 91128, France

**Keywords:** bioinspiration, fish fins, shape morphing, architected materials, soft robotics

## Abstract

Morphing materials are typically either very compliant to achieve large shape changes or very stiff but with small shape changes that require large actuation forces. Interestingly, fish fins overcome these limitations: fish fins do not contain muscles, yet they can change the shape of their fins with high precision and speed while producing large hydrodynamic forces without collapsing. Here, we present a ‘stiff’ morphing beam inspired from the individual rays in natural fish fins. These synthetic rays are made of acrylic (PMMA) outer beams (‘hemitrichs’) connected with rubber ligaments which are 3–4 orders of magnitude more compliant. Combinations of experiments and models of these synthetic rays show strong nonlinear geometrical effects: the ligaments are ‘mechanically invisible’ at small deformations, but they delay buckling and improve the stability of the ray at large deformations. We use the models and experiments to explore designs with variable ligament densities, and we generate design guidelines for optimum morphing shape (captured using the first moment of curvature), that capture the trade-offs between morphing compliance (ease of morphing the structure) and flexural stiffness. The design guidelines proposed here can help the development of stiff morphing bioinspired structures for a variety of applications in aerospace, biomedicine or robotics.

## 1. Introduction

**Figure 1.**
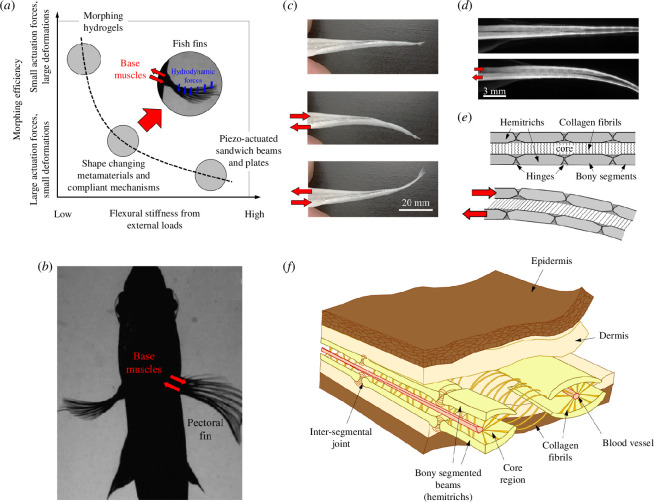
Key features in individual fin rays (adapted from [15]). (*a*) Fish fins combine high morphing efficiency and high stiffness from external loads, two properties that are mutually exclusive in engineering morphing materials. (*b*) An individual ray from a fish fin harvested from Atlantic salmon (*Salmo salar*) is ‘morphed’ by applying push/pull forces manually. (*c*) Micro-CT scans of a caudal fin ray from Atlantic salmon in rest position and actuated position. The hemitrichs ‘glide’ on one another, which induces flexural deformations.(*d*) Fish can change the shape and effective surface of their fins from actuation by base muscles (adapted from [16]). (*e*) Cross-section of a fin showing the bony rays and surrounding compliant tissues. (*f*) Schematic of this mechanism.

Morphing refers to radical shape changes in materials and structures [1], which involve large amplitudes and/or unusual deformation modes such as auxetics [2] or the coupling of deformation modes such as compression-induced twist [3]. When a change of geometry is desired in an engineering system, morphing materials offer more advantages than traditional discrete mechanisms: lighter weight, smoother transitions, better distributed stresses, simpler kinematics, smaller numbers of actuators and higher reliability. A wide range of technologies are available for engineering morphing materials: metamaterials [4–6], origami [7], kirigami [8], hydrogels [9], hygromorphs [10] and pneumatic shape-morphing elastomers [11,12]. Radical shape change is achieved in these materials, but only with relatively compliant materials and structures that cannot sustain large external forces without excess deformations, collapse or failure. Stiffer and stronger structures can also be morphed using piezoelectric actuators [13] or shape memory alloys [14], but they require large actuation forces that result in only relatively small morphing amplitudes. This conflict between ‘morphing efficiency’ and stiffness from external loads, illustrated in figure 1*a*, has been a major obstacle to the systematic use of morphing materials in aerospace and other domains [17]. Other design strategies have been proposed for morphing structures, with interesting combinations of stiffness and morphing. However, these structures rely on complex micro-architectures found by topology optimization [18] on local instabilities [19] or on multi-part mechanisms with actuation that must be transmitted through the entire structure, involving pulleys [20], sliding elements [21], shafts [22] or pneumatics [23] covered by flexural skin, or complicated architected materials made of hundreds of moving parts [22,24].

Interestingly, fish fins from ray-finned fishes (*Actinopterygii*) seem to overcome these limitations. Fish fins do not contain muscles but display large morphing amplitudes, combined with high stiffness from external loads (hydrodynamic forces), fast response times and actuation from the base only. Fish fins ‘probably represents the most elaborate and refined adaptation to efficient interaction with water that has ever evolved’ [25] and as such, they can serve as models for the design of new morphing materials. Because they contain no muscles, fish fins are often thought of as passive swimming surfaces which are simply ‘flapped’ for propulsion or passive stabilization. Fish fins are in fact much more sophisticated systems: fish can adjust not only the orientation, but also the curvature, shape and surface of their fins to finely tune hydrodynamic interactions and to generate powerful forces in three dimensions [16] (figure 1*b,c*). Individual fish fins are composed of a collagenous membrane stiffened by 10–30 beam-like structures called rays. Each ray has a diameter in the order of ~100 μm with a tapered profile and aspect ratio > 100 (figure 1*d–f*). The rays are composed of two bony layers called hemitrichs which are connected by collagen fibrils embedded in a ground gel-like substance (figure 1*f*).

A remarkable feature of fish fins is that their curvature can be adjusted solely by muscular actuation from the base of the rays (figure 1*b*). Push/pull actuation induces shear deformations in the core region, while rotations at the base are prevented by the configuration of the tendons and by a cartilaginous pad at the base of the fin [16]. The shear deformation imposed at the base induces a competition between flexural deformations of the hemitrichs and the shear deformations of the compliant core over the length of the fin. There is a fine balance between the flexural stiffness of the hemitrichs and the shear stiffness of the core, so that individual rays can morph along their entire length [26]. Individual rays must also be stiff to minimize deformations and prevent collapse when subjected to hydrodynamic loads. Flexural experiments on individual rays have indeed revealed relatively high flexural stiffness, with homogenized flexural modulus in the order of 1 GPa [27]. For comparison, synthetic materials with similarly large morphing amplitudes are orders of magnitude more compliant. Responsive hydrogel hybrids, for example, have an elastic modulus in the order of only 0.1–1 MPa [9]. We have recently measured and modelled the morphing and flexural stiffness performance of individual rays from rainbow trout (*Oncorhynchus mykiss*) [28]. We found that the collagenous core region is best modelled with spring elements that duplicate the arrangement of the collagen fibrils. We also found by fitting mechanical experiments that these elements are 3–4 orders of magnitude more compliant than the hemitrichs. To properly capture the large deformations and large rotations of natural rays, linear models used previously for fins [15,16,26,29,30] are not adequate, and numerical models that capture geometrical nonlinearities are required.

Fish fins represent an elegant, robust and mechanically efficient solution to stiff morphing, and they have already inspired a multitude of morphing structures: three-dimensional printed segmented composites [15], flexible fish-like robotic systems [31–37], aquatic–aerial vehicles or robots [38–40], bioinspired morphing fins as propulsors and to improve manoeuvrability for autonomous underwater vehicles (AUVs) [41–48]. Fish fins have also inspired a variety of robotic gripper designs [49–52]. However, among these fish-inspired structures, few truly duplicate the mechanisms of morphing of the natural rays. For example, a majority of fin-inspired robots simply rely on passive, flexural membranes. A thorough understanding of the interplay between compliant and stiff elements at regimes of large deformations and large rotation is still needed.

Here, we propose a new design for a fin-inspired stiff morphing beam that captures the main features of natural rays: stiff outer hemitrichs connected by compliant ligaments, morphing induced by push–pull forces at the base resulting in large deflections and large rotations. We explore the mechanics of morphing and flexural deflection of these rays using combinations of mechanical experiments and nonlinear finite element (FE) models, which ultimately leads to design guidelines for fin ray-inspired morphing beams.

## 2. Synthetic fin ray: design and fabrication

**Figure 2.**
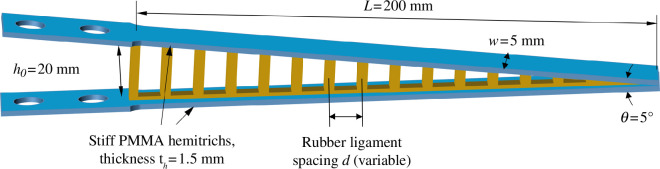
Overview of the design and geometry for the synthetic ray.

Individual fin rays are complex three-dimensional structures (figure 1), and the objective of this study was to focus only on some of the main structural features: (i) a slender and slightly tapered overall geometry; (ii) a pair of stiff hemitrichs connected with compliant, elastic ligaments; and (iii) hemitrichs at least three orders of magnitude stiffer than the ligamentous core region. Figure 2 shows a design that captured these key features. The overall dimensions of the ray (*L* = 200 mm by *h_0_* =20 mm by *w* = 5 mm) and the taper angle (*θ* = 5°) were maintained constant throughout this study, to focus on the effects of the relative properties of hemitrichs and core region. The outer hemitrichs were made from 1.5 mm thick polymethyl methacrylate sheets (PMMA; US Plastic, Lima, OH, USA). PMMA is a relatively stiff polymer, with a measured flexural modulus *E_h_* = 2.8 GPa. Each hemitrich is a *t_h_* = 1.5 mm thick and *w* = 5 mm wide beam, with a wider region at the base for attachment to our mechanical testing platform. To duplicate the softer collagenous core in natural rays we used 1.6 mm thick rubber sheets (RubberCal, Santa Ana, CA, USA; figure 2). The collagenous core in natural rays has a fibrillar structure, with collagen fibrils aligned perpendicularly to the axis of the ray (figure 1*b*), a fibrillar arrangement with important implications for the mechanical performance of the ray. For this study, we maintained the cross-section of the individual ligament constant (1.6 mm by 1.6 mm), but we varied their spacing *d* (figure 2) to manipulate the effective elastic properties of the core region. The homogenized, effective tensile modulus *E_c_* of the core was then calculated using:


(2.1) $$E_{c}=\frac{E_{r}A_{r}}{dw},$$

where *E_r_* is the tensile modulus of rubber (measured *E_r_* = 1.52 MPa), *A_r_* is the cross-section of individual ligaments (*A_r_* = 2.56 mm^2^) and *w* is the width of the hemitrich (*w* = 5 mm). Different designs were created and fabricated by varying the distance *d* between the ligaments from 1.6 to 16.6 mm, resulting in an effective tensile modulus of the core region *E_c_* in the 50–500 kPa range. The contrast in elastic properties between the PMMA hemitrichs and the ligamentous rubber core was therefore more than three orders of magnitude, consistent with measurement on natural fin rays [28].

**Figure 3.**
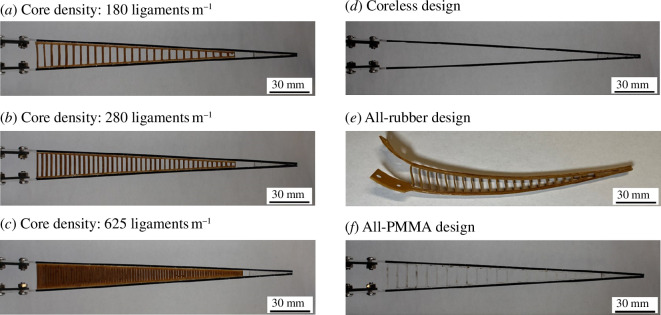
Examples of synthetic fin rays: (*a–c*) Fin rays with ligaments of increasing density; (*d*) coreless fin ray (i.e. no ligaments connect the hemitrichs); (*e*) an ‘all-rubber’ design; and (*f*) ‘all-PMMA’ design.

Rubber needs to be ‘preconditioned’ in order to settle its microstructure and produce repeatable results across repeated cycles of loading and unloading [53,54]. Therefore, prior to fabrication, we manually stretched the rubber sheets by about 50% strain for 10 cycles. The hemitrichs and rubber ligaments were then cut from sheets using an 80W CO_2_ precision laser cutter (Nova35; Thunder Laser Systems, Houston, TX, USA). Laser cutting produced components with high dimensional fidelity, and we verified that the dimensions of the laser cut components (hemitrich width, ligaments cross-section and spacing) were as specified and repeatable using an optical microscope (Leica DM2700 M). The two hemitrichs were glued together at the tip using cyanoacrylate and 5° angle wedges to control the taper angle of the ray accurately. The ligamentous core was then glued to the inner surfaces of the two hemitrichs using cyanoacrylate, and the fin ray was then allowed to cure for at least 24 h. Using this fabrication procedure, we fabricated fin rays with nine different ligament spacings ranging from *d* = 1.6 to *d* = 16.6 mm, corresponding to ligament densities of about 180–630 ligaments m^−1^ (figure 3*a–c*). We also fabricated three ‘extreme’ designs: a ‘coreless’ design with no ligaments (figure 3*d*), a ray entirely made of rubber (figure 3*e*) and a ray entirely made of PMMA (figure 3*f*).

## 3. Mechanical testing

**Figure 4.**
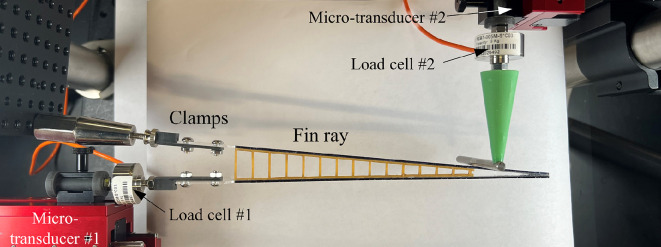
Experimental platform used for the morphing test and the flexural test on synthetic fin rays.

The synthetic rays were tested using the micromechanical testing platform shown in figure 4. The upper hemitrich was clamped to the base of the set-up, while the lower hemitrich was clamped in line with a motorized micromanipulator (SOLO Single Axis Manipulator Controller, Sutter Instrument, Novato, CA, USA). The first type of test was the morphing test, where the lower transducer imposed a displacement *u_0_* on the base of the lower hemitrich, while the resulting actuation force *F_0_* was recorded with a precision load cell (REB7 Subminiature Load Cell, 5 kg capacity, Loadstar Sensors) mounted in line with the transducer. The second type of test was the flexural test, where both hemitrichs were clamped. A transverse displacement *δ* was imposed at a distance *L_s_* = 130 mm from the base, and the corresponding transverse force *P* was recorded with a precision load cell mounted in line with the transducer. For both tests, optical images of the ray were automatically acquired at regular intervals using a digital camera (Canon EOS Rebel T6). The control of the transducers, the acquisition of the forces and the acquisition of the pictures were all managed with an interface and a unified custom Matlab code. The morphing and flexural tests were non-destructive, so each ray could be tested multiple times in either morphing or flexural configuration, with highly repeatable results. At the post-processing stage, the images were analysed using a custom Matlab code to track the position of the hemitrich and digitally reconstruct the deformed shape (‘elastica’). To this end, the sides of the hemitrichs were painted in black.

**Figure 5.**
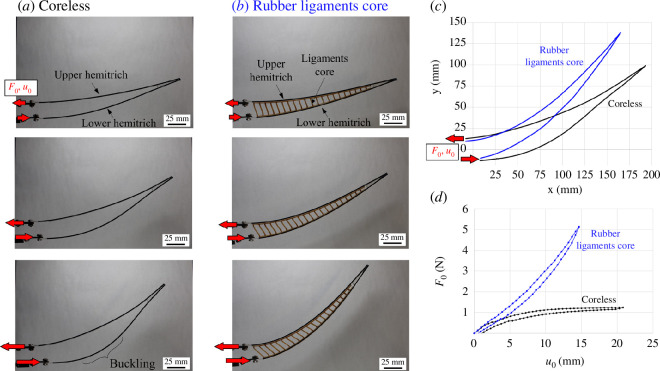
Typical results from morphing experiments: snapshot of morphed rays at three levels of imposed actuation displacement *u_0_* for (*a*) rays with no core (coreless) and (*b*) rays with rubber ligament cores (130 ligaments m^−1^); (*c*) comparison of morphed profiles (elastica); (*d*) comparison of actuation force–displacement (*F*_*0*_–*u*_*0*_) curves for coreless fin ray (black) and ray with rubber ligamentous core region (blue).

Figure 5 shows typical results from morphing tests on a fin ray with a ligament spacing *d* = 7.6 mm, shown together with results from a ‘coreless’ ray for reference (i.e. no ligaments). At small deformations, both designs display the same mechanical response, because the ligaments, initially perpendicular to the hemitrichs, produce little resistance to morphing (they are ‘mechanically invisible’). At larger deformations, however, there are pronounced differences between the responses of these two designs. The coreless design shows evidence of buckling of the lower hemitrich (which carried a compressive force), accompanied by softening on the *F_0_–u_0_* curve, while the design with ligaments shows stiffening and a much more stable response.

**Figure 6.**
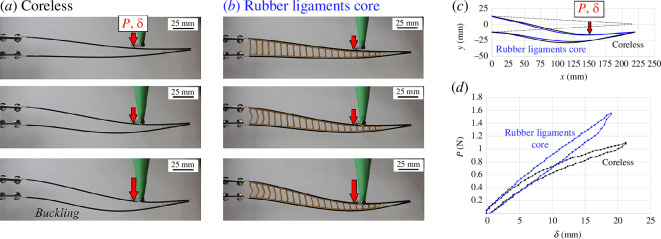
Typical results from flexural experiments: snapshot of deflected rays at three levels of imposed deflection *δ* for (*a*) rays with no core (coreless) and (*b*) rays with rubber ligament cores (130 ligaments m^–1^); (*c*) comparison of profiles (elastica); (*d*) comparison of transverse force–displacement (*P–δ*) curves for coreless fin ray (black) and ray with rubber ligamentous core region (blue).

Figure 6 shows typical results from the cantilever test on the same two rays. Here again, the two designs produce identical mechanical responses at small deformations, where the contribution of the ligament to overall stiffness is negligible. At larger deformations, the coreless design again shows evidence of buckling in hemitrichs, while the design with a core displays a more stable mechanical response and sustained stiffness. We also observed that the ligaments buckle at large deformations, mainly due to the loading nose applying a transverse compression on the core region.

These results highlight an important function of the ligaments: the ligaments are mechanically ‘invisible’ at small deformations, which is beneficial because it decreases the actuation force required to initiate morphing from the straight configuration. At large deformation, however, the ligaments rotate and stretch, stiffening the entire structure. Importantly, they keep the hemitrichs together at large deformations, which increases the actuation force but also delays the buckling of the hemitrichs. The ligaments also enable a more thorough morphing of the ray in terms of deformation and kinematics. We finally note that while all curves presented here show an elastic response where the initial shape of the ray was recovered upon removal of the load, there is also a small amount of hysteresis from viscous dissipation in the hemitrichs and ligaments.

## 4. Finite element model and design exploration

**Figure 7.**
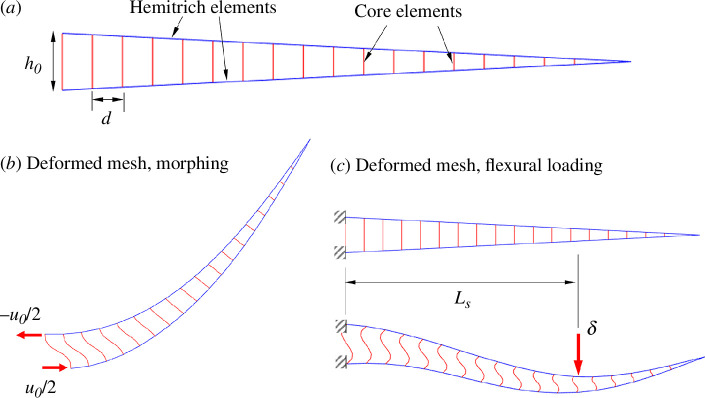
Nonlinear FE model: (*a*) Mesh with nonlinear corotational beam element: stiff blue elements for the hemitrich and softer red elements for the ligaments; boundary conditions and typical deformed shapes for (*b*) morphing and (*c*) flexural loading.

In order to model and optimize the mechanics of the ray, we developed a nonlinear FE model that captures large deformations and large rotations [28] (figure 7). The core nonlinear algorithm as well as the model input and output were coded on an in-house developed Matlab code [27]. The hemitrichs were modelled with elastic corotational beam elements, with all stiffnesses derived from the modulus of PMMA and the cross-sectional dimensions of the hemitrichs. In the model, these elements are predominantly deformed in flexion. The ligaments were modelled explicitly, using their cross-section and the modulus of rubber. These elements were predominantly deformed in tension, although some bending was also observed.

**Figure 8.**
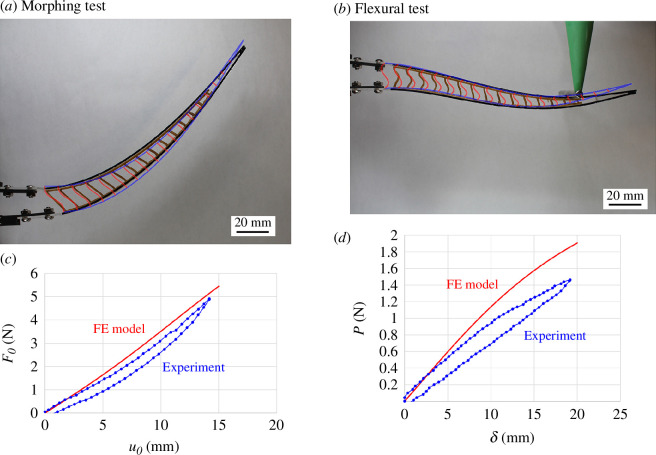
Comparison between experiments and models: (*a*) elastica and (*c*) *F_0_–u_0_* curves from pure morphing test; (*b*) elastica and (*d*) *P–δ* curves from the flexural test.

Figure 8 shows comparisons between the model predictions and the experiments. Experiments and FE models for the morphing and flexural loading configurations agree well in terms of deformed shapes (figure 8*a,c*). The FE models captured the nonlinear response of the ray for morphing and flexural deformations. They however overestimated the flexural stiffness and the morphing stiffness by up to about 15% (figure 8*b,d*), which we attributed to imperfections in the physical model (in particular, the onset of buckling in the experiments may be reduced by defects which were not included in the models). Nevertheless, this model provided a robust and computationally efficient approach to modeling the ray-like beam at large deformations.

The design of this bioinspired morphing structure has several key parameters, which can be tuned and optimized using the models and experiments described above. However, it is important to first define what constitutes a ‘good design’ for this structure. For this study, we used three performance metrics: the morphing curvature, which captured whether the deformation due to morphing propagated along the entire ray; the morphing compliance, which captured how much ‘actuation’ force is needed to morph the ray; and the flexural stiffness which characterized the stiffness of the structure when subjected to an external transverse force. These three metrics are described in more detail in the following sections.

### 4.1. Morphing curvature

**Figure 9.**
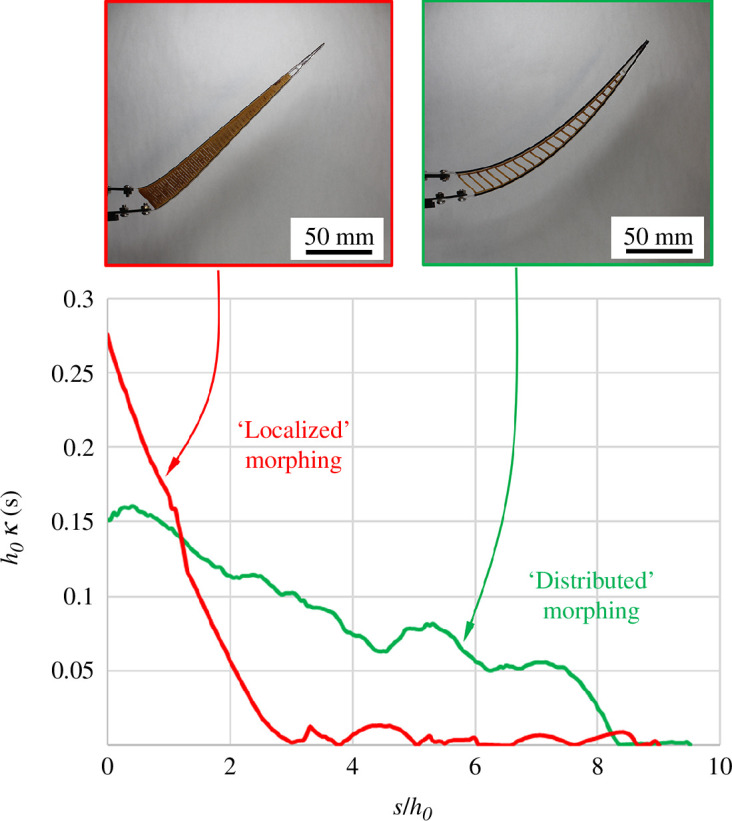
Normalized local curvature as a function of curvilinear position along the ray. The red curve shows an example of ‘localized morphing’ where the deformations are concentrated near the base of the ray, which is not desirable. The green curve is an example of ‘distributed morphing’ where curvature from morphing is better distributed along the length of the ray.

When subjected to actuation at the base, the curvature of natural fin rays is distributed almost over their entire length, in other words, fish can manipulate not only the orientation but also the shape of their fins solely from muscular push–pull at the base of the fins. Our morphing experiments on the synthetic fins produced a similar response but to various extents. An important measure of the morphing shape in this configuration is the local curvature of the ray κ(s) , where *s* is the curvilinear position from the base of the ray (note that curvature κ is the inverse of the radius of curvature, so that for a straight, undeformed ray *κ* =0). We measured κ(s) from the images acquired during the experiments, and also from the nodal positions in the model, in both cases using the Pratt method [55]. Figure 9 shows two plots of κ(s) normalized by *h_0_* as a function of the curvilinear distance *s* from the base (also normalized by *h_0_*). In one of the designs, the density of ligaments is high, which produces a stiff core region and a curvature concentrated near the base (figure 9). This type of response may be described as a deformation hinge near the base of the ray, while the rest of the ray rotates but remains undeformed. In the second case, the density of ligaments is lower, which produces a more compliant core region and a morphing response that is well distributed over the entire length of the ray. In terms of morphing, the second case is preferable, but a metric is needed to quantitatively rank and optimize different designs.

In a recent study on morphing lattice beams [56], we examined several possible metrics for flexural morphing, all based on curvature. The maximum curvature $\kappa_{max}$ and an average curvature $\bar{\kappa }$ (the curvature averaged over the length of the ray) were first considered as morphing metrics, but these metrics are not adequate: cases where the deformations are concentrated near the base of the ray, forming a deformation ‘hinge’ represent poor morphing response but produce high values of these metrics (the very high curvature near the base of the ray biasing $\kappa_{max}$ and $\bar{\kappa }$ towards high values). A better metric is the first moment of curvature *κ^(1)^* given by [56]:


(4.1) $$\kappa^{\left(1\right)}=\frac{1}{L}\int_{0}^{L}s\kappa \left(s\right)ds.$$

**Figure 10.**
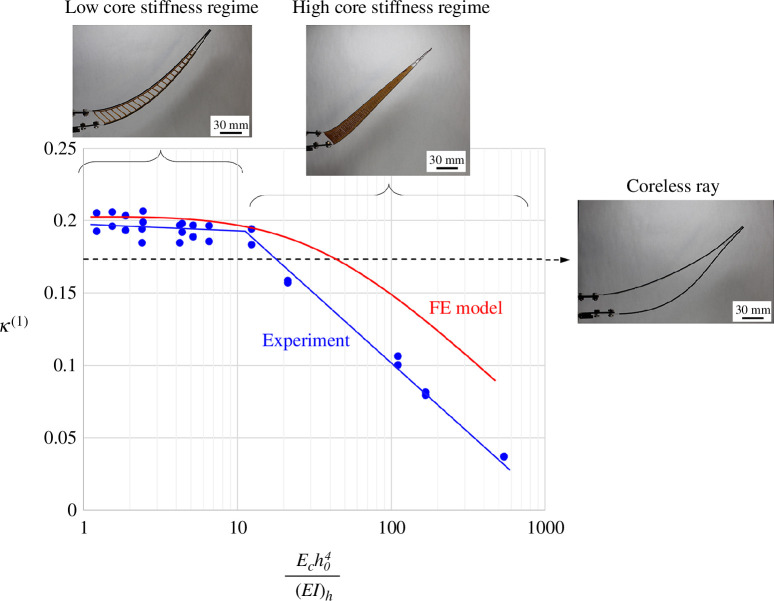
First moment of curvature $\kappa^{\left(1\right)}$ as a function of relative core modulus across fin rays with different core spacings. Typical snapshots for the different regimes are also shown.

Large curvatures away from the base of the ray produce *κ* ^(1)^ values, and therefore *κ* ^(1)^ captures morphing that occurs over long distances along the ray. *κ* ^(1)^ is a robust, non-dimensional metric of the flexural morphing of the ray in terms of deformations, which can be used to compare and rank different fin ray designs. Figure 10 shows *κ* ^(1)^ as a function of the relative stiffness of the core, obtained from experiments and the nonlinear FE model for an actuation distance *u_0_* = 13 mm. The reference is the coreless ray ( $E_{c}=0$ ). For the coreless case, buckling of the compressive hemitrich impedes morphing and limits *κ* ^(1)^. Both the model and the experiments show a similar trend: the addition of ligaments keeps the hemitrichs together and adds stability, leading to higher values for *κ* ^(1)^ (*κ* ^(1)^ ~ 0.2), but only in the range of relative core thickness $1<\frac{E_{c}h_{0}^{4}}{{\left(EI\right)}_{h}}<10$ . For higher relative core stiffness $\frac{E_{c}h_{0}^{4}}{{\left(EI\right)}_{h}}>10$ , *κ* ^(1)^ decreases, because as the core region becomes too stiff, morphing becomes concentrated near the base. In summary, the ligaments in the core are critical to stabilize the hemitrichs and prevent buckling, but too many ligaments and a high relative core stiffness lead to a hinge-type response (figure 9) which is not desired.

### 4.2. Morphing compliance

**Figure 11.**
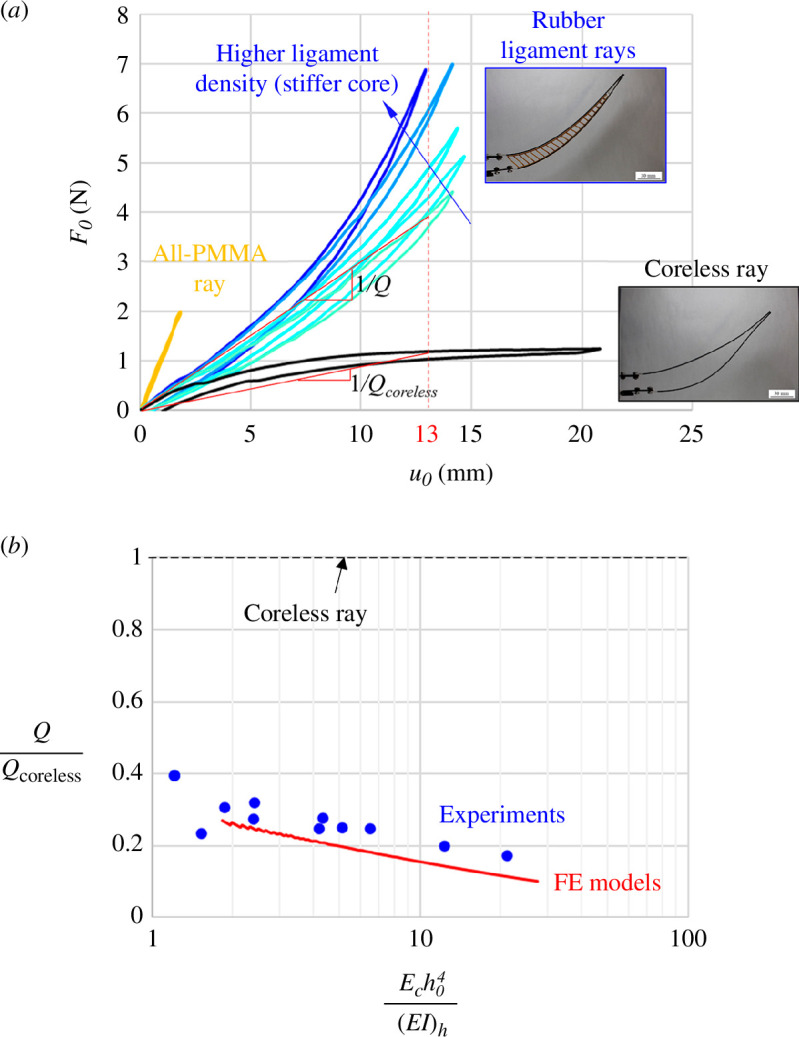
(*a*) *F_0_–u_0_* curves for different synthetic fin rays. The actuation stiffness increases (and the compliance *Q* decreases) for stiffer core stiffness *E_c_*. The coreless ray shows softening associated with buckling. (*b*) Morphing compliance, normalized by the morphing compliance of the coreless design (*E_c_* = 0) from models and experiments.


Another metric of interest is the amount of actuation force required to morph the ray. Figure 11*a* shows a set of representative *F_0_–u_0_* curves (actuation force as a function of actuation distance) for different designs. The coreless design and the designs with ligaments show the same initial morphing stiffness, because at small deformations, the ligaments are mechanically invisible. There are however pronounced differences at higher deformations as nonlinear mechanisms become pronounced. In the coreless design, the buckling of one of the hemitrichs leads to a softening *F_0_–u_0_* response. For the design with ligaments, the progressive stretching and large rotations of the ligaments stabilize the ray but it also leads to a stiffening response similar to what we observed on the natural ray [28]. As expected, the amount of stiffening is more pronounced as the ligament density is increased. For comparison, we also show the results from the all-PMMA ray (figure 3*e*), which produces a stiff linear response restricted to small deformations (high deformations broke the ray). To quantitatively compare these designs, we use the morphing compliance *Q* to capture the ‘ease’ of morphing the ray from a set of forces at the base. It is simply written as:

(4.2) $$Q=\frac{u_{0}}{F_{0}},$$

where *u_0_* is the base actuation displacement and *F_0_* is the actuation force at the base of the ray. By this metric, the different designs only show differences at large deformations, for which the response is nonlinear. For this reason, we used the secant modulus of the *F_0_–u_0_* curves, taken at an actuation distance of *u_0_* = 13 mm for all designs. Figure 11*b* shows the morphing compliance, normalized by the morphing compliance of the coreless design, as a function of relative core stiffness. Both experiments and FE models show a similar trend: the designs with ligaments are 2.5–5 times less compliant than the coreless design (they require 2.5–5 times more force to morph). As expected, the morphing compliance decreases for stiffer relative core stiffnesses.

### 4.3. Flexural stiffness

**Figure 12.**
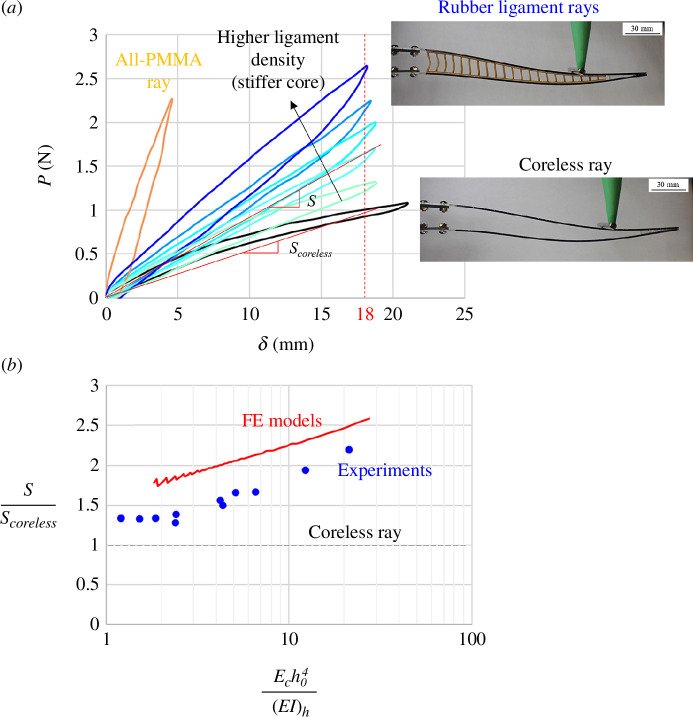
(*a*) *P-δ* curves for different synthetic fin rays. The secant flexural stiffness increases for stiffer core stiffness *E_c_*. The coreless ray shows softening associated with buckling. (*b*) Flexural stiffness, normalized by the morphing compliance of the coreless design (*E_c_* = 0) from models and experiments.

Natural fins can be morphed to large amplitudes, but they also need to be relatively stiff to produce and sustain hydrodynamic forces without collapsing. A third metric we considered for our synthetic rays is flexural stiffness, which we measured as the stiffness of the ray when subjected to a transverse force. Figure 12*a* shows representative transverse force–deflection curves (*P-δ* curves) for different designs. The coreless design and the ligament designs all show the same initial flexural stiffness, again because the ligaments are mechanically invisible at small deformations. At larger deformations, the curves soften because of buckling. However, the results clearly show that buckling can be delayed by increasing the density of the ligaments. An all-PMMA representative *P-δ* curve is also shown, which shows a much stiffer response but which is restricted to small deformations. In order to measure the flexural stiffness of the ray in a way that accounts for large deformation regimes, we used a secant modulus taken at *δ* = 18 mm for all designs. Figure 12*b* shows the secant flexural stiffness, normalized by the flexural stiffness of the coreless design, as a function of the relative stiffness of the core. Even the lowest density of ligament increases the flexural stiffness by about 40%, by delaying buckling and from $\frac{E_{c}h_{0}^{4}}{{\left(EI\right)}_{h}}\approx 2-3$ , the flexural stiffness continuously increases with relative core stiffness. The FE models overestimate the experiments by about 10–20%, but they properly capture the trend of flexural stiffness vs. relative core stiffness.

### 4.4. Multi-objective plots

**Figure 13.**
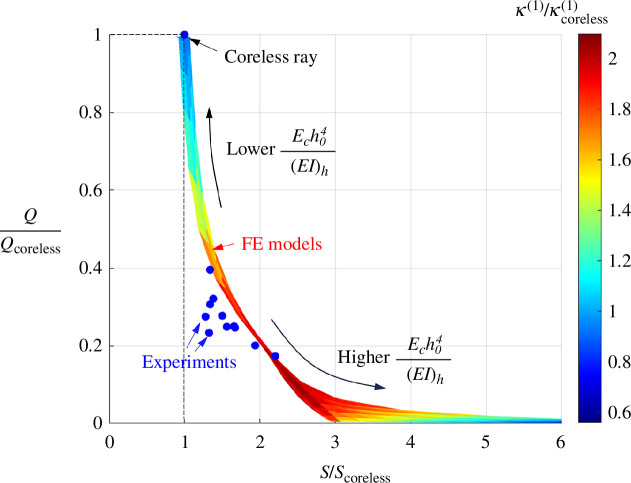
Performance map showing the morphing compliance *Q*, the flexural stiffness *S* and the first moment of curvature *κ* ^(1)^, from FE models and from experiments. All properties are normalized by the properties of the coreless design.

The ideal ray combines high morphing first moment of curvature *κ* ^(1)^, high morphing compliance *Q* and high flexural stiffness *S*. However, the results above suggest that some of these performance metrics are conflicting. For example, higher ligament densities increase flexural stiffness but also decrease morphing compliance. To visualize these trade-offs, it is useful to plot these metrics on a performance map, which we constructed by considering different possible combinations of core modulus *E_r_* and ligament spacing *d* in the 1–20 mm range for the FE models. All three metrics were then normalized by the performance of the coreless design to create the map shown in figure 13. The possible combinations of flexural stiffness and morphing compliance all lie on a narrow region on the map, which is largely governed by the relative core stiffness parameter $\frac{E_{c}h_{0}^{4}}{{\left(EI\right)}_{h}}$ . A closer examination of the model revealed that the ‘thickness’ of that region is due to the flexural stiffness of the ligaments, which has a much smaller effect than the relative core stiffness, but which must be accounted for in the model to match the experiments (especially for designs with larger ligament densities). As expected, the coreless ray produces the highest morphing compliance, but the lowest flexural stiffness. As the ligaments are added and their density is increased, $\frac{E_{c}h_{0}^{4}}{{\left(EI\right)}_{h}}$ increases, with the effect of decreasing the morphing compliance and increasing the flexural stiffness. The map therefore clearly shows the trade-off between these two properties. On the other hand, the morphing metric *κ* ^(1)^, shown as colour-coded on the map of figure 13, shows a clear optimum region on the map within the range $1<\frac{E_{c}h_{0}^{4}}{{\left(EI\right)}_{h}}<10$ producing the highest values. The experimental results for various designs, also shown in figure 13, agree well with the model, with deviation up to a maximum of about 15%.

We finally consider the parameter *C* = *Q*.S , which must be maximized to combine high morphing compliance and high flexural stiffness. We use the data of figures 5, 6 and 13 to estimate *C* ~ 10^−1^ for our synthetic fins. The parameter *C* is also useful to compare our synthetic fish fins with other existing morphing materials reported in the literature. The mechanical forces and stiffness are not always fully characterized in existing morphing materials, and the values greatly depend on dimensions and configurations. Nevertheless, *C* can be estimated using simple assumptions and models. We consider here a *L* = 200 mm beam with cross-section *h* × *h* = 400 mm^2^. The flexural stiffness of the beams is then estimated using *S*~ $Eh^{4}/L^{3}$ , with the modulus *E* dependent o the material of which the structure is made.

The first example we used for comparison is a very soft morphing structure: pneumatic morphing systems that include inflatable fabrics [57] and pneumatic cells [58]. In these materials, the modulus is of the order of *E* ~ 100 kPa, and the actuation compliance can be estimated using $Q\approx \lambda L/P_{a}A$ , where *P_a_* is the actuation air pressure (typically of the order of 0.1 MPa), *A* is the cross-section of the structure and *λ* is the stretch ratio of the pressurized cells (typically *λ* ~ 1.5). Using these estimates, we calculated *C* = *Q*.S ~ 10^−4^ to 10^−2^ for pneumatic morphing systems. On the other end of the stiffness range for morphing materials, we examined stiff structural materials (*E* ~ 100 GPa) actuated with piezoelectric shear actuators [59]. For this class of morphing materials, the actuation force can be estimated using *EAd_p_e*, where *d_p_* is the piezoelectric coefficient (*d_p_* ~ 10^12^ mV) and *e* is the strength of the electric field. Using these assumptions, we estimated *C* ~ 10^−2^ for piezoelectric-actuated stiff structures. For comparison, our synthetic fins produced *C* ~ 10^−1^, which suggests that morphing beams inspired from rays provide advantageous combinations of low actuation forces and high flexural stiffnesses compared to existing morphing technologies.

## 5. Conclusion

Fish fins are fascinating structures that are relatively stiff, but which can be morphed from relatively small push–pull forces applied at their base. In particular, the individual rays that compose the fins can serve as models for improved stiff morphing engineering materials, but further development is slowed by the lack of mechanical models at regimes of large deformations, and by the lack of guidelines for design and optimization. In this study, we have designed, fabricated and tested synthetic fin rays that duplicate some of the key features of natural fish fin rays. We used a combination of experiments and nonlinear FE models to reach the following main conclusions:

— The combination of a very soft material (rubber) with a ligamentous design enables core regions which are 3–4 orders of magnitude more compliant than the hemitrichs.
— The morphing and deformation mechanisms observed on natural fin rays could be duplicated in our synthetic rays.
— The ligamentous architecture in the core region is critical so that the ligaments are ‘mechanically invisible’ at small deformations, which minimizes the force required to initiate morphing.
— The ligamentous design in the core region is critical to stabilize the structure at large deformations, by delaying the buckling of the hemitrichs in both morphing and flexural loading configurations.
— The stiffness of the core region can be easily tuned by changing the spacing between ligaments.
— The ligaments are required to achieve morphing deformations distributed over the entire ray, which we measure using the first moment of curvature. The ligaments provide a constant morphing shape over a wide range of stiffnesses. However, if the stiffness of the core is too high, morphing localizes in a ‘hinge’ near the base of the ray, so that the morphing shape is optimum only for a certain range of stiffness in the core region.
— The main parameter that governs the mechanics and performance of the ray is the ratio between the tensile stiffness of the core and the flexural stiffness of the hemitrichs. The flexural stiffness of the ligament has much smaller effects, but it must be accounted for to match the experiments, especially at high ligament densities.
— A property map showing all three metrics can be used to guide the design of ray-like morphing beams. There is a strong trade-off between morphing compliance (the actuation forces required to morph the ray) and flexural stiffness, but there is a clear region where the morphing shape is optimum.

The structure of our synthetic rays captured some of the main features of natural fin rays, which are remarkably uniform across all species of ray-finned fishes (*Actinopterygii*) [60]. There are however variations in morphologies and functionalities across species [29,61], which could be examined in the context of bioinspiration, to possibly improve engineering designs further. In addition, there are many possible extensions and enrichments of the design that could incorporate some finer features inspired from natural fin rays (figure 1). Segmentations in the hemitrichs could be added to the design to achieve better trade-offs between morphing compliance and flexural stiffness [15]. The section of the hemitrich also decreases towards the end of the rays, which creates a gradient of flexural properties that could also improve the mechanical performance of the rays. Other finer features in natural rays include semi-cylindrical three-dimensional bony segments in the hemitrichs, crimps in the collagen fibrils and non-uniform distributions of ligament spacings. The mechanical role of these features is not fully understood, but the fabrication, testing and modelling platforms we developed here could serve as mechanical models to better understand these more complex structure–property relationships in natural fins. These additional features could also lead to even better stiff morphing bioinspired structures for a variety of applications in aerospace, biomedicine or robotics.

## Data Availability

The authors confirm that the data supporting the findings of this study are available within the article.
